# Distribution and Habitat Modeling of *Diploknema butyracea* Under Past, Present, and Future Climatic Conditions

**DOI:** 10.1002/ece3.72412

**Published:** 2025-10-25

**Authors:** Shreehari Bhattarai, Ripu M. Kunwar, Arjun K. Shrestha, Balram Bhatta, Binaya Adhikari

**Affiliations:** ^1^ Faculty of Forestry Agriculture and Forestry University Hetauda Nepal; ^2^ Gandaki University Pokhara Nepal; ^3^ Faculty of Agriculture Agriculture and Forestry University Rampur Nepal; ^4^ Department of Biology University of Kentucky Lexington Kentucky USA

**Keywords:** bioclimatic variables, climatic niche, habitat suitability, spatial niche, species distribution model

## Abstract

Climate change has profoundly impacted global weather patterns, intensifying extreme events and shifting seasonal trends. Species distribution models (SDMs) are crucial for assessing ecological effects, particularly in forecasting climate‐induced shifts. This study examined the distribution of *Diploknema butyracea* by refining and spatially thinning occurrence data to a 1 km resolution, reducing an initial 413 records to 80 unique localities. Nineteen bioclimatic variables from the CHELSA dataset were analyzed, retaining only those with variance inflation factor (VIF) values below 10. Climatic conditions from past, present, and future scenarios were incorporated to assess habitat suitability over time. To ensure model reliability, block partitioning cross‐validation was applied, identifying the LQ model (Rm = 1.5) as the most effective. Key determinants of habitat suitability included bio08 (mean temperature of the wettest quarter), bio06 (minimum temperature of the coldest month), bio07 (temperature annual range), bio12 (annual precipitation), and bio18 (precipitation of the warmest quarter). During the Last Glacial Maximum (~20,000 years ago), suitable habitats were largely confined to the southern lowlands of Nepal. By the mid‐Holocene, rising temperatures and increased precipitation enabled expansion into mid‐hill regions. The present distribution shows high suitability across mid‐hills and lowlands, while future projections (SSP3‐7.0) indicate declining suitability in the lowland Terai due to increasing temperatures and precipitation, with a slight increase at higher elevations. These findings underscore the importance of climatic stability in shaping the distribution of *D. butyracea* and offer valuable insights for conservation planning and forest restoration in the face of climate change.

## Graphical abstract

This study analyzed the distribution of * *Diploknema butyracea* * using species distribution models (SDMs) to assess climate‐induced shifts over time. Key climatic factors influencing habitat suitability included temperature and precipitation variables, with historical trends showing expansion from lowlands to mid‐hills. Future projections (SSP370) indicate declining suitability in lowland Terai due to rising temperatures and precipitation, highlighting the need for conservation planning amid climate change.

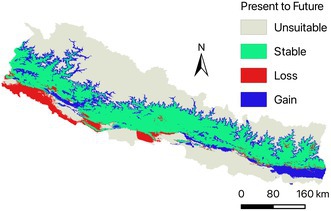

## 1 Introduction

Climate change has significantly impacted global weather systems and is expected to continue altering seasonal patterns (Hajek and Knapp 2022), increasing extreme weather events, and causing temperature and precipitation fluctuations (Kunwar et al. 2023; Vincze et al. 2017). The ongoing burning of fossil fuels and ecosystem degradation will likely intensify these changes, leading to irreversible effects on ecosystems, biodiversity, species, habitats, and distribution ranges (Muluneh 2021; Shrestha et al. 2021; Tse‐ring et al. 2010). Consequently, species distribution shifts, habitat fragmentation, and extinction are expected to increase (Colwell et al. 2008). Predicting these abrupt changes remains challenging due to uncertainties in climate modeling (Hallegatte et al. 2018). Species distribution models (SDMs) are vital tools in ecological studies, particularly in assessing threats from invasive species (Nagendra et al. 2013) and predicting climate change impacts on species distribution (Booth 2018). Among SDMs, the Maximum Entropy (MaxEnt) model has proven highly accurate, especially when species distribution data are incomplete (Yi et al. 2017). MaxEnt has been widely used in identifying suitable habitats for endangered species, assessing climatic suitability, and prioritizing conservation efforts (Lu et al. 2012; Koch et al. 2017; Zheng et al. 2016).

*Diploknema butyracea* (Roxb.) H. J. Lam, commonly known as the Butter tree (Chiuri in Nepali), is a multipurpose species from the Sapotaceae family (Joshi 2010). It is a latex‐yielding tree distributed across tropical regions and economically significant genera such as *Diploknema*, *Manilkara*, *Pouteria*, *Chrysophyllum*, and *Madhuca* (Ke et al. 1996; Press et al. 2000). *Diploknema* is a native genus to Southeast Asia, the Himalayas, and southwestern China, comprising seven species, with Nepal harboring only *D. butyracea* (Press et al. 2000). It is indigenous to the sub‐Himalayan regions of Nepal, India, China, and Bhutan and thrives at elevations between 200 and 1800 m (Joshi 2010; Lee and Pennington 1996; Majumdar et al. 2012; Press et al. 2000).

It has played a crucial role in the livelihoods of ethnic groups across Nepal, India, Bhutan, and Tibet (Adhikari‐Devkota et al. 2023). In Nepal, various ethnic groups have utilized the tree and its parts for centuries for sustenance and cultural practices (Adhikari‐Devkota et al. 2023; Bhattarai et al. 2021; Manandhar 2002). The tree holds deep cultural significance in Chepang ethnic groups, where it is often given as dowries and regarded as a symbol of pride (Chikanbanjar et al. 2021; Dhakal 2014; Uprety and Asselin 2023). The Chepang people, a semi‐nomadic and marginalized ethnic minority, predominantly inhabit the steep terrains of central Nepal, particularly in Chitwan, Makawanpur, Dhading, Gorkha, and Tanahun districts (CBS 2021; Gurung 1989; Rijal 2011). Additionally, Chiuri butter is commonly used for lighting lamps in Buddhist temples, while the seeds hold monetary value as “Dakshina” in religious rituals (Paudel and Wiersum 2002). Beyond its cultural and economic importance, Chiuri contributes significantly to biodiversity, recreation, and the household economy, earning recognition as “Kalpabriksha”, a wish‐fulfilling sacred tree (Bhattarai et al. 2021; Dhakal 2014; Joshi et al. 2018). However, despite its multifaceted value, the species is under threat due to habitat degradation, deforestation, overexploitation, invasive plant encroachment, and the impact of climate change (MoFSC 2014). To ensure the conservation of this valuable species, this study aims to develop a model for its distribution and habitat suitability using MaxEnt and ArcGIS tools to analyze the climatic suitability of Chiuri in Nepal.

## 2 Materials and Methods

### 2.1 Species Occurrence Records, and Environmental Data

**FIGURE 1:**
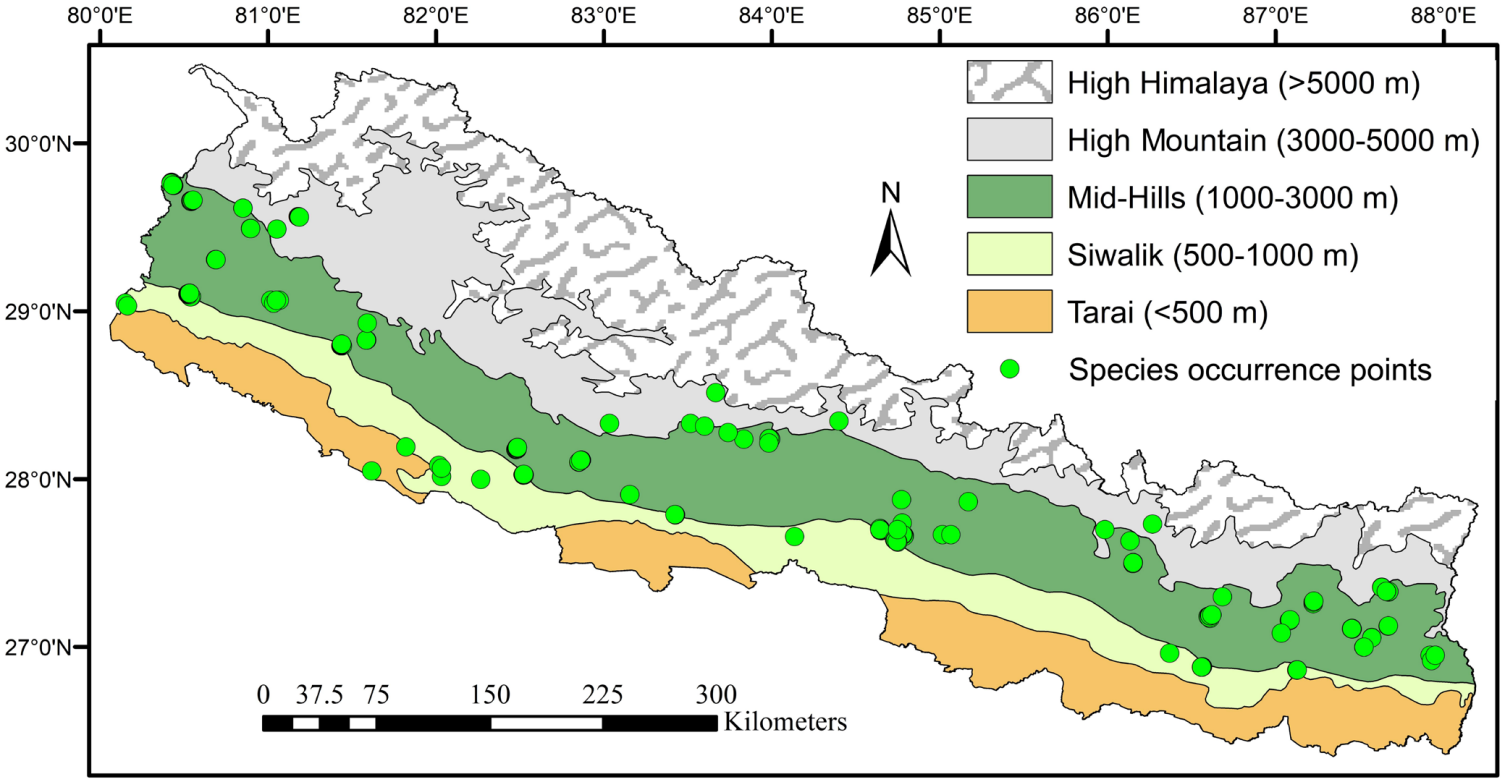
Study area map showing physiographic regions of Nepal and the distribution of occurrence points of *Diploknema butyracea* .

The study was conducted across Nepal, a region with diverse climatic zones ranging from subtropical lowlands to alpine highlands (Figure 1). Administratively, Nepal is divided into seven provinces, 77 districts, and 753 local bodies, whereas it has five distinct vertical physiographic regions from north to south: (i) High Himalaya (above 5000 m) with 24% area, (ii) High Mountains (3000–5000 m) with 20% area, (iii) Mid‐Hills (1000–3000 m) with 30% area, (iv) Siwalik (500–1000 m) with 12% area, and (v) Tarai (< 500 m) with 14% area (MoSTE 2014). Occurrence data for *D. butyracea* were cleaned and spatially thinned to a 1 km resolution, resulting in 80 unique localities from an initial 413 records that were primarily collected throughout the country (Figure 1), while a few occurrence points were gathered from herbarium deposited at National Herbarium (KATH), Royal Botanic Garden K, and GBIF data set. A total of 300 localities were removed for sharing the same grid cell. Background data comprised 10,000 random pseudo‐absence points sampled within the study extent, excluding cells containing known presence, to represent environmental availability across Nepal following standard MaxEnt practice (Phillips et al. 2006).

**FIGURE 2:**
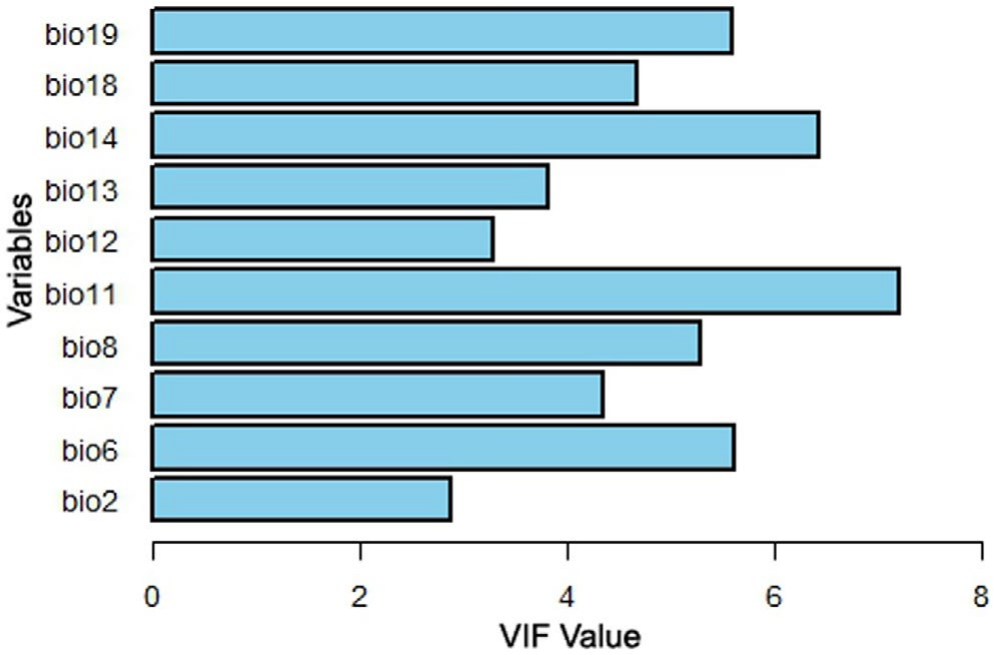
Variance Inflation Factor (VIF) value of the environmental variables.

A set of 19 biologically relevant bioclimatic variables (bio1–bio19) (Table 1) was sourced from the CHELSA (Climatologies at High Resolution for the Earth's Land Surface Areas) at a spatial resolution of 30 arc‐seconds (approximately 1 km^2^) (Zheng et al. 2016; Xu et al. 2019). These variables capture essential temperature and precipitation gradients relevant to the ecological niche of *D. butyracea* . To address high collinearity among the bioclimatic variables, we calculated the Variance Inflation Factor (VIF) following methods outlined by Rana et al. (2020) and Fox and Weisberg (2011). This process, implemented using R version 3.4.1 (R Development Core Team version 2024.04.0), facilitated the removal of redundant, highly correlated variables, which can obscure the influence of predictors in ensemble modeling approaches (Ranjitkar et al. 2014). The final set of explanatory variables was determined by retaining only those with VIF values less than 10 (Figure 2). Variables with VIF ≥ 10 (e.g., bio01, bio04) were excluded to avoid multicollinearity. Retained predictors (VIF < 10) represented distinct climatic niches, with bio08, bio06, and bio12 showing the strongest ecological relevance for *D. butyracea* .

**TABLE 1:** List of environmental variables used in the model development.

Code	Environmental variables	Unit
bio1	Annual mean temperature	°C
bio2	Mean diurnal range (Mean of monthly (max temp‐min temp))	°C
bio3	Isothermality (bio2/bio7*100)	%
bio4	Temperature seasonality (standard deviation × 100)	°C
bio5	Max temperature of warmest month	°C
bio6	Min temperature of coldest month	°C
bio7	Temperature annual range (bio5–bio6)	°C
bio8	Mean temperature of wettest quarter	°C
bio9	Mean temperature of driest quarter	°C
bio10	Mean temperature of warmest quarter	°C
bio11	Mean temperature of coldest quarter	°C
bio12	Annual precipitation	mm
bio13	Precipitation of wettest month	mm
bio14	Precipitation of driest month	mm
bio15	Precipitation seasonality (coefficient of variation)	1
bio16	Precipitation of wettest quarter	mm
bio17	Precipitation of driest quarter	mm
bio18	Precipitation of warmest quarter	mm
bio19	Precipitation of coldest quarter	mm

Past and future climatic scenarios were incorporated to evaluate habitat suitability over time. For the Past Climate Projections, Paleoclimatic data from the TraCE‐21 k project were used to reconstruct conditions during two distinct periods: the Last Glacial Maximum (LGM; ~20,000 years ago) and the mid‐Holocene (~6000 years ago). For Future Climate Projections, Bioclimatic variables for 2071–2100, representing the late 21st century, were used. These projections were derived from the CHELSA dataset and based on the MPI‐ESM1‐2‐HR global climate model under the SSP3‐7.0 scenario, which depicts a medium‐to‐high emission scenario characterized by regional competition and limited mitigation efforts.

### 2.2 Maxent Modeling for Past and Future Prediction, and Change Analysis

Species distribution modeling was performed using MaxEnt within the Wallace ecological modeling platform, employing various feature classes and regularization multipliers to estimate habitat suitability based on environmental predictors. Among the configurations tested—Linear (L), Linear‐Quadratic (LQ), Hinge (H), Linear‐Quadratic‐Hinge (LQH), and Linear‐Quadratic‐Hinge‐Product (LQHP)—the optimal model was identified as LQ with a regularization multiplier (Rm) of 1.5. This configuration demonstrated the best balance between predictive accuracy and complexity, as indicated by the lowest delta AICc value of 0, signifying superior fit compared to other models evaluated. The selection of LQ with Rm 1.5 underscores its suitability for accurately predicting species habitat distribution under the studied environmental conditions. We assumed a “full dispersal” scenario that is, species can colonize any climatically suitable grid cell, so these projections represent potential climatic suitability rather than actual range shifts constrained by barriers such as mountain ridges and seed‐dispersal limits.

### 2.3 Model Evaluation and Threshold Selection

Model robustness was evaluated through cross‐validation using a block partitioning method, incorporating multiple metrics to ensure reliability and accuracy. Delta AICc was employed to identify the most parsimonious model by balancing model fit and complexity. At the same time, the Area Under the Curve (AUC) metric assessed the model's discriminatory performance for both training and validation datasets. Prediction accuracy was further evaluated using omission rates at the minimum training presence threshold (OR_mtp) and the 10% omission threshold (OR_10p). The Boyce Index (CBI) was also used to measure the reliability of predictions. A p10 threshold of 0.364 was applied to generate binary habitat suitability maps, effectively excluding the lowest 10% of predicted suitability values to refine the model's outputs.

## 3 Results

### 3.1 Model Performance

The LQ with Rm = 1.5 model emerged as the optimal choice based on comprehensive evaluation metrics. It achieved a Delta AICc of 0, signifying the most parsimonious configuration among all tested models. Regarding discriminatory power, the model demonstrated a mean validation AUC of 0.712, indicating good performance in distinguishing between presence and absence points. The omission rates further underscore its effectiveness: OR_mtp stood at 0.15, indicating high accuracy in capturing occurrence points, while OR_10p at 0.4375 suggests a balanced approach between omission and commission errors. Moreover, the Boyce Index yielded a mean validation CBI of 0.274, highlighting moderate reliability in model predictions. Overall, these findings validate the robustness and efficacy of the LQ with Rm = 1.5 model in ecological modeling contexts.

### 3.2 Environmental Variable Contributions and Response Curves

**FIGURE 3:**
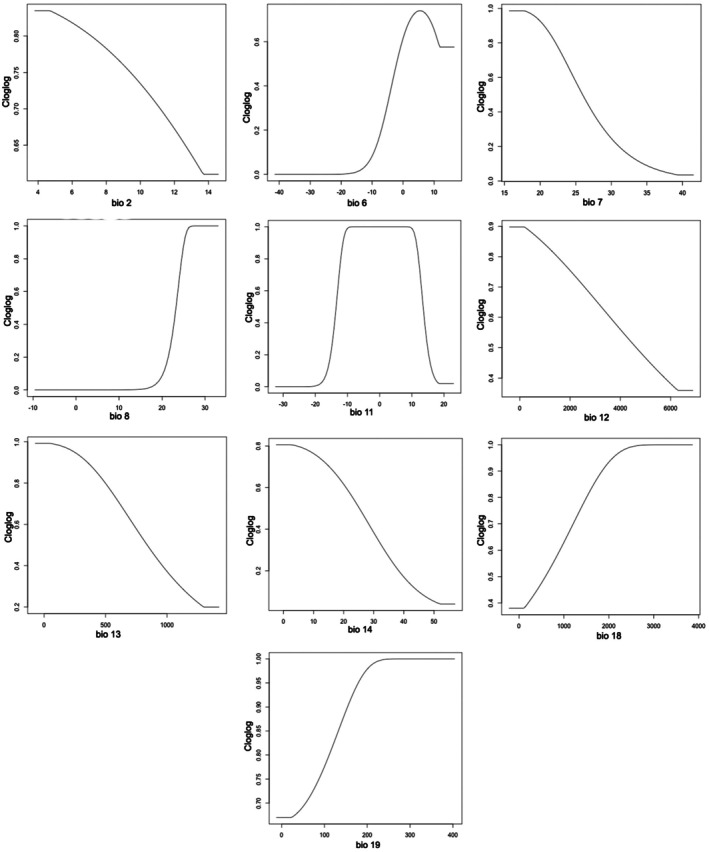
Response curves of environmental variables to distribution probability.

Based on the MaxEnt lambda file, the most influential predictors of habitat suitability for *D. butyracea* were identified as bio08 (Mean Temperature of Wettest Quarter), bio06 (Minimum Temperature of Coldest Month), bio07 (Temperature Annual Range), bio12 (Annual Precipitation), and bio18 (Precipitation of Warmest Quarter) (Figure 3). Among these, bio08 had the highest positive influence, with suitability increasing sharply as temperatures during the wettest quarter rose, likely plateauing beyond an optimal threshold. Bio06 also showed a positive relationship, with habitat suitability increasing with warmer minimum temperatures during the coldest month, peaking around 10°C. Conversely, bio07 exhibited a negative effect, with suitability decreasing as annual temperature variability increased, suggesting a preference for thermally stable environments. Similarly, bio12 negatively impacted suitability, indicating that excessive annual precipitation reduced habitat favorability. In contrast, bio18 positively influenced suitability, with regions experiencing higher precipitation during the warmest quarter identified as more suitable. These predictors collectively highlight the species' preference for regions with moderate rainfall, warmer winters, and stable thermal conditions.

### 3.3 Temporal Patterns of Habitat Suitability

**FIGURE 4:**
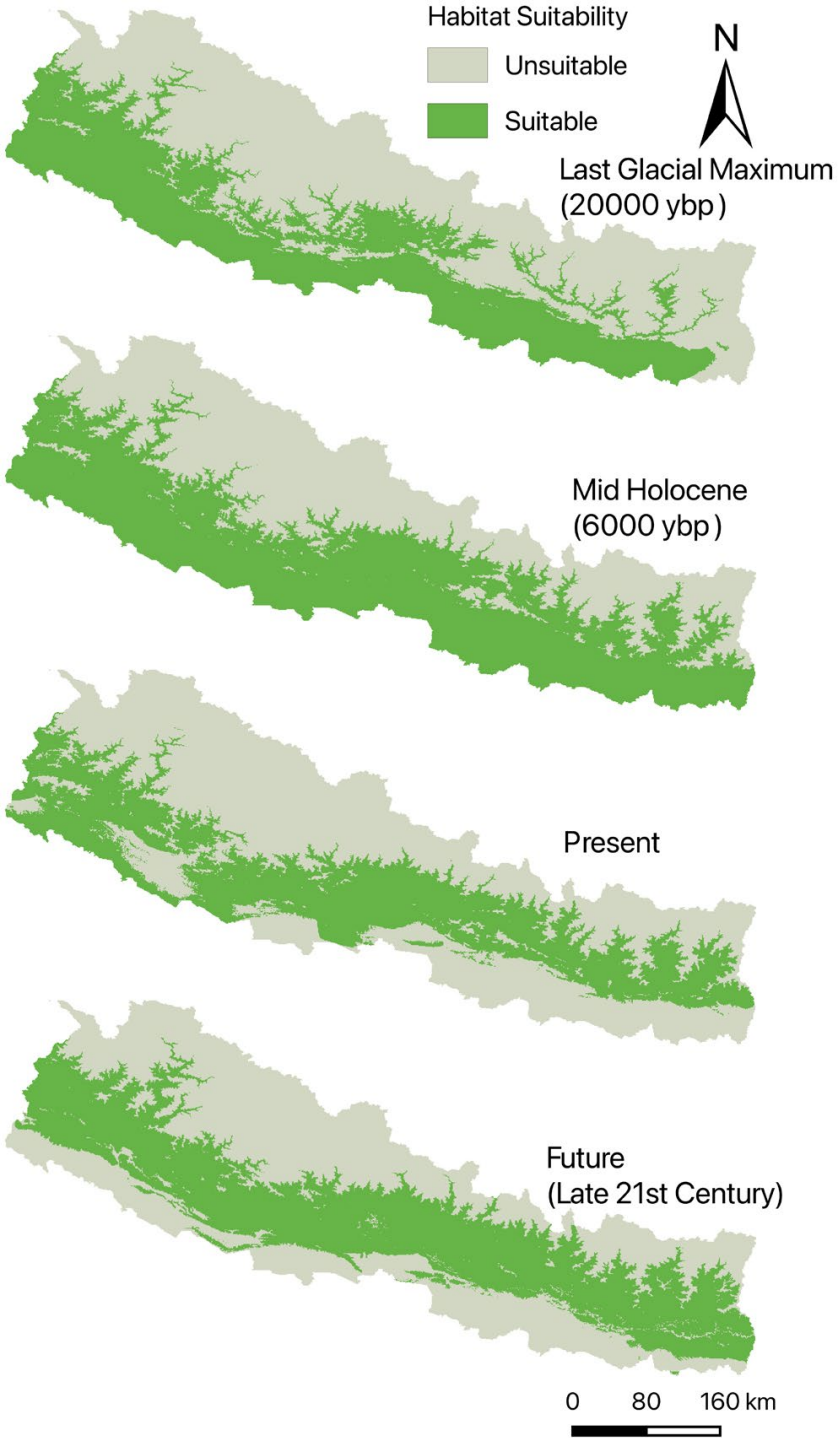
Temporal patterns of habitat suitability.

**FIGURE 5:**
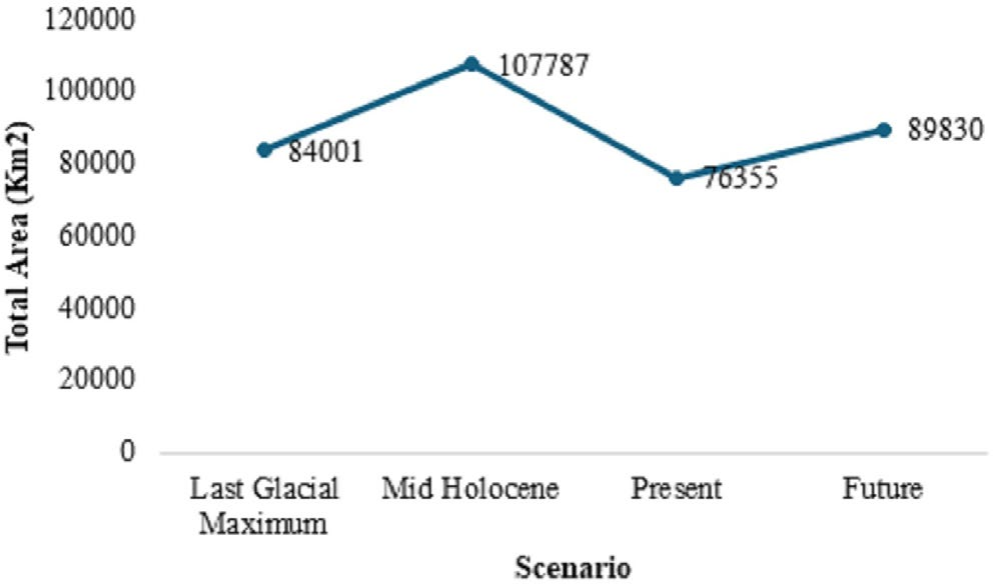
Suitable area of *D. butyracea* under different climatic scenarios.

The habitat suitability maps for *D. butyracea* reveal distinct temporal patterns across four time periods: the Last Glacial Maximum (LGM, ~20,000 years before), the mid‐Holocene (~6000 years before), the present, and the late 21st century (2071–2100) (Figure 4). These projections provide insights into the species' historical and future distribution trends based on climatic changes (Figure 5).

Last Glacial Maximum (LGM, ~20,000 ybp): During the LGM, habitat suitability was primarily restricted to the southern lowland regions of Nepal, with minimal suitability in the mid‐hills and high‐altitude zones. This pattern reflects the colder and drier conditions of the LGM, where the species likely favored warmer microclimates and less extreme environmental variability.

Mid‐Holocene (~6000 ybp): By the mid‐Holocene, habitat suitability expanded significantly into the mid‐hill regions, corresponding to the relatively warmer and wetter conditions of this period. This expansion likely represents a period of increased ecological stability and favorable climatic conditions for the species.

Present: The current distribution shows the widest habitat suitability across Nepal, with high suitability throughout the mid‐hills and parts of the lowland regions. This pattern aligns with the species' ecological preference for moderate precipitation, stable temperatures, and thermally buffered environments, as identified in the response curve analysis.

Future (Late 21st Century, 2071–2100): Future projections under the SSP3‐7.0 scenario indicate a contraction of habitat suitability in the lowland Terai region due to increasing temperatures and precipitation, while mid‐hill regions remain suitable. However, the suitability in higher altitudes is expected to increase slightly, potentially reflecting a shift toward cooler areas in response to warming trends.

These temporal patterns highlight the dynamic nature of *D. butyracea* 's habitat suitability in response to climatic changes. The contraction in lowland suitability and the persistence of mid‐hill regions underscore the species' sensitivity to extreme temperature and precipitation conditions. This trend is consistent with the importance of bio06 (Minimum Temperature of Coldest Month), bio08 (Mean Temperature of Wettest Quarter), and bio07 (Temperature Annual Range), which were identified as key predictors in the modeling process. Collectively, these findings emphasize the potential for climate‐induced range shifts, necessitating targeted conservation strategies to ensure the persistence of suitable habitats for *D. butyracea* in Nepal.

### 3.4 Habitat Stability From Present to Future

**FIGURE 6:**
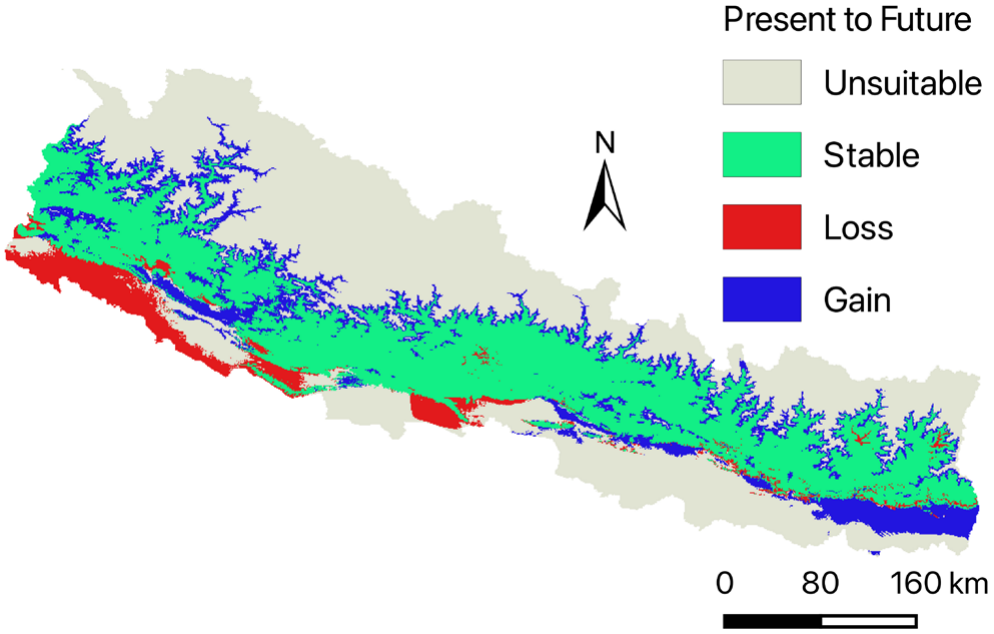
Suitable habitat of distribution from the present to the future.

The map illustrating habitat dynamics from the present to the future shows three primary categories: stable habitat, habitat loss, and habitat gain (Figure 6). Stable habitats, represented in green, dominate the mid‐hill regions of Nepal, indicating these areas are likely to remain suitable under future climatic conditions. Habitat loss, depicted in red, is concentrated in the lowland Terai region, reflecting increasing unsuitability due to projected climatic changes. Conversely, habitat gain, shown in blue, is observed in higher altitudes, suggesting a potential upward range shift of *D. butyracea* as the species adapts to warmer future conditions.

### 3.5 Stable Habitat Across Climatic Time Frames

**FIGURE 7:**
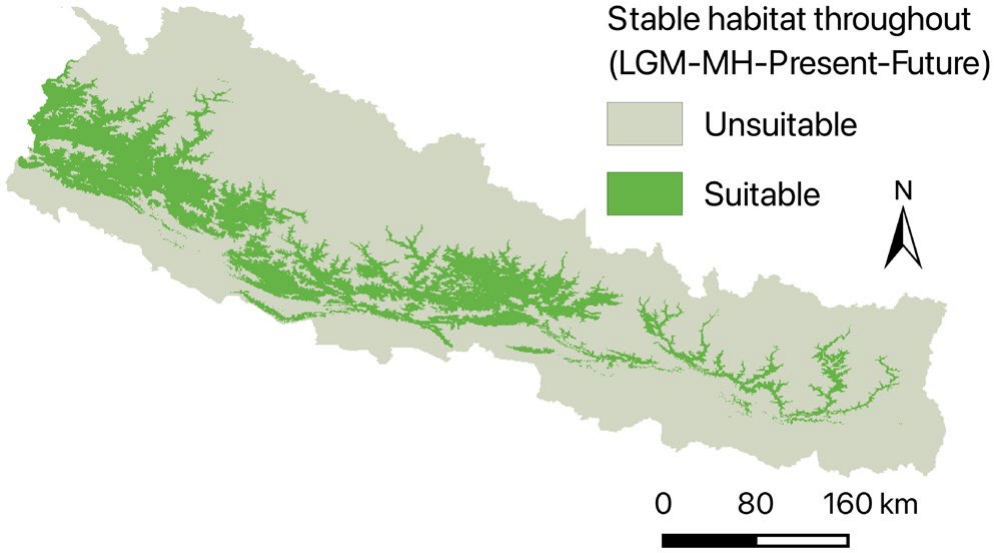
Stable habitat throughout the temporal patterns.

The map depicting stable habitat across all four climatic time frames (LGM, mid‐Holocene, Present, and Future) reveals regions that have consistently provided suitable conditions for *D. butyracea* . These stable areas, marked in green, are primarily concentrated in the mid‐hills, spanning the central and eastern regions of Nepal (Figure 7). This stability underscores the resilience of these habitats to climatic fluctuations over time. The identification of these long‐term stable habitats provides crucial insights for conservation planning, as these areas represent refugia that have historically supported the species and are likely to remain critical for its survival in the future.

## 4 Discussion

Many studies utilize 19 bioclimatic variables from the WorldClim Global Climate Data as crucial environmental factors in the MaxEnt model to predict species distributions, abundance patterns, and the spread of invasive species (Pearson and Dawson 2003; Xu et al. 2019). Nevertheless, incorporating edaphic and biogeographic factors would enhance the model's accuracy and reliability (Ranjitkar et al. 2014). Forecasting the climatic niche of species is vital for habitat conservation and sustainable management in the future (Profirio et al. 2014). Nevertheless, some studies overlook the redundancy introduced by highly correlated variables, which adversely affects the reliability of prediction results (Duflot et al. 2018). Factors with low contribution rates and high correlations were effectively eliminated, leading to the selection of 10 key environmental variables and the reconstruction of the model, resulting in improved prediction accuracy. The model achieved a mean validation AUC of 0.712 within the “fair” to “good” discrimination range and a Boyce Index of 0.274, indicating moderate reliability. The concordance of AUC, Boyce, and low omission rates (> 85% of presence captured) demonstrates robust discrimination and sensitivity. Habitat suitability also reflects key regional‐local gradients, being shaped by both broad‐scale climate and fine‐scale geographic factors in mountain environments (Beniston 2003; Kerr and Dobrowski 2013; Korner 2007). Extensive field sampling likely further enhanced model performance (Convertino et al. 2011). We recommend that future work further report complementary metrics (e.g., TSS, partial ROC) to provide a more comprehensive assessment of predictive accuracy.

Temperature‐related variables rather than precipitation variables were more influential variables in determining the habitat suitability. The strong positive influence of bio08 (mean temperature of the wettest quarter) suggests that warmer temperatures during the wettest months enhance habitat suitability, likely due to improved physiological and growth conditions. Similarly, bio06 (minimum temperature of the coldest month) exhibited a positive relationship, indicating that *D. butyracea* thrives in areas where winter temperatures remain relatively mild. This aligns with the species' preference for warmer climates, which may facilitate seed germination and overall survival as observed in other important medicinal plants (Rana et al. 2020). Conversely, bio07 (temperature annual range) showed a negative effect, suggesting that high annual temperature fluctuations negatively impact habitat suitability. This preference for thermal stability implies that extreme temperature variations could pose physiological stress on the species, affecting its distribution. Likewise, the negative influence of bio12 (annual precipitation) indicates that excessive rainfall may create unfavorable conditions, potentially due to waterlogging or increased competition with other vegetation types. However, bio18 (precipitation of the warmest quarter) positively contributed to suitability, highlighting the importance of sufficient moisture availability during the growing season. Collectively, these findings suggest that *D. butyracea* favors regions with moderate precipitation, warmer winters, and stable thermal conditions, emphasizing the critical role of climatic stability in shaping its distribution.

Our finding of high‐elevation suitability gains aligns with Zu et al. (2021), who documented upward migration of subtropical plants in the eastern Himalayas (e.g., *Taxus wallichiana*). Similar to our results, Zu et al. (2021) reported a 15%–20% expansion in suitable habitat at higher elevations (> 1500 m) under RCP4.5, underscoring a regional trend of climate‐driven range shifts. The projected decline in Terai suitability mirrors observations for *Dactylorhiza hatagirea* by Shrestha et al. (2021), who attributed losses to warming and erratic rainfall. This parallels Shrestha et al. (2021)'s findings for medicinal herbs in Nepal, where lowland habitats became unsuitable under SSP scenarios due to heat stress. The persistence of mid‐hill refugia matches Kunwar et al. (2023)'s multi‐species analysis, identifying 1000–2000 m as a climate‐resilient zone. Kunwar et al. (2023) likewise highlighted mid‐elevation forests as critical refugia for endemic species under climate change, supporting our prioritization of these areas for conservation.

The temporal shifts in habitat suitability for *D. butyracea* across bioclimatic periods highlight its sensitivity to changing conditions. During the Last Glacial Maximum (LGM), its restricted southern lowland range suggests a reliance on warmer microclimates amid generally cold, dry conditions, while the Mid‐Holocene expansion into mid‐hills reflects more favorable warmth and moisture. The present distribution, with high suitability in both mid‐hills and lowlands, aligns with the species' preference for moderate precipitation and stable temperatures. However, our results show the present potential climatic niche is the smallest (Mid‐Holocene>Future > LGM>Present), driven by increased temperature seasonality and reduced dry‐season precipitation under the current climate. Under SSP3‐7.0, future warming (projected +3°C–6.3°C) and ~30% higher precipitation by the 2090s (NCVST 2009; Salick et al. 2009) partially offset these effects at higher elevations but still do not restore the broader Mid‐Holocene optimum.

The dominance of stable habitats in the mid‐hill regions suggests that these areas will continue to provide suitable environmental conditions, reinforcing their importance for long‐term conservation. However, the significant habitat loss in the lowland Terai region indicates that rising temperatures and changing precipitation patterns will make these areas increasingly unsuitable. This contraction aligns with the species' sensitivity to extreme climatic conditions, particularly excessive heat and precipitation variability. Meanwhile, the observed habitat gain in higher altitudes suggests a potential upward migration, likely driven by the need for cooler and more stable conditions as the climate warms, as also predicted for other valuable medicinal plants (Rana et al. 2017). This upward shift is consistent with ecological responses observed in many montane species adapting to global climate change (Aryal 2015; Zu et al. 2021). These findings highlight the necessity of planning conservation strategies such as indigenous knowledge‐based conservation, awareness, local stewardship, agroforestry practices, including monitoring potential areas for emerging suitable habitats and protecting mid‐hill forests to maintain stable populations.

Beyond these broad elevational trends, we observe notable east–west contrasts across Nepal. The wetter Koshi Zone in the east likely buffers climate impacts, aiding habitat retention, whereas the drier, more seasonal Karnali Basin in the west exacerbates suitability losses. Moreover, steeper, fragmented terrain in western Nepal may further impede upslope colonization compared to gentler eastern slopes. Because our SDM omits non‐climatic factors such as soil properties, land‐use change, and dispersal barriers, future work should integrate high‐resolution topographic variables (e.g., slope, aspect) and connectivity analyses to explain these regional disparities. Nonetheless, the core patterns of mid‐hill stability, Terai contraction, upslope gains, and east–west variability in potential climatic suitability provide a robust baseline for targeted conservation planning and further ecological investigation.

Sensitivity and uncertainty analysis in species distribution modeling is crucial for evaluating model performance. Such analyses enable local authorities to make quantitative, species‐specific decisions by simulating various management scenarios under different environmental conditions (Rengstorf et al. 2013). Edaphic factors and climatic variables play a fundamental role in shaping species distribution patterns. Furthermore, a thorough assessment of observed distribution trends about predicted climatic conditions across different periods can serve as a foundation for informed decision‐making, conservation strategies, and resource management. Understanding the relationship between species and their environment is essential for examining ecological requirements and spatial distribution patterns (Yi et al. 2018).

The MaxEnt model, while advantageous due to its ease of use and generally high prediction accuracy, has limitations in prediction precision. It assumes an ideal ecological niche without considering key factors such as altitude, species self‐diffusion ability, species interactions, vegetation types, soil composition, human activities, and geomorphological characteristics (Xu et al. 2019). Additionally, it does not account for demographic pressures, including deforestation, fringe forest interference, fire, logging, overgrazing, intensive agriculture, shifting cultivation, or natural calamities in the northwestern Himalayas. The species in this region often exist in isolated and scattered populations across mountain cliffs, hindering gene flow through pollen dispersal during fertilization, while recalcitrant seeds that are difficult to germinate further exacerbate conservation challenges. Furthermore, high human population density near forested areas and environmental changes significantly impact species dispersal. Despite these limitations, species distribution modeling remains a valuable tool for future conservation and management efforts.

## 5 Conclusions

This study underscores the importance of bioclimatic variables, particularly temperature‐related factors such as bio08 and bio06, in determining the habitat suitability of *D. butyracea* . The model projections align with observed climate‐driven range shifts, including upward elevational migration and the critical role of mid‐hill refugia, consistent with trends seen in other Himalayan species. These patterns illustrate the dynamic response of habitat suitability to projected climate shifts, emphasizing the need to concentrate conservation efforts in stable regions while also considering areas with potential habitat expansion for future management. The projected distribution changes of Chiuri follow the trend: mid‐Holocene > Future > Last Glacial Maximum > Present. This study examines the impact of climate change on Chiuri's ecological niche in the Nepal Himalayas under a medium‐to‐high emission scenario, characterized by regional competition and limited mitigation efforts. Overall, the findings provide essential baseline data for evaluating climate change effects on the species while advocating for broader‐scale adaptive strategies in forest tree restoration and conservation planning, considering the uncertainties of future climate conditions.

## Author Contributions

**Shreehari Bhattarai:** conceptualization (lead), data curation (lead), formal analysis (lead), methodology (lead), writing – original draft (lead), writing – review and editing (lead). **Ripu M. Kunwar:** conceptualization (equal), investigation (equal), methodology (equal), supervision (lead), validation (equal), writing – review and editing (equal). **Arjun K. Shrestha:** conceptualization (equal), investigation (equal), methodology (equal), supervision (lead), writing – review and editing (equal). **Balram Bhatta:** conceptualization (equal), investigation (equal), methodology (equal), supervision (lead), writing – review and editing (equal). **Binaya Adhikari:** formal analysis (equal), investigation (equal), methodology (equal), supervision (equal), writing – review and editing (equal).

## Conflicts of Interest

The authors declare no conflicts of interest.

## Supporting information


**Data S1:** ece372412‐sup‐0001‐DataS1.xlsx.

## Data Availability

All the required data are uploaded as Supporting Information.

## References

[ece372412-bib-0001] Adhikari‐Devkota, A. , J. Pandey , and H. P. Devkota . 2023. “ *Diploknema Butyracea* (Roxburgh) HJ Lam.” In Himalayan Fruits and Berries. Academic Press.

[ece372412-bib-0002] Aryal, P. 2015. “Climate Change and Its Impact on Medicinal and Aromatic Plants: A Review.” Climatic Change 1: 49–53.

[ece372412-bib-0003] Beniston, M. 2003. “Climatic Change in Mountain Regions: A Review of Possible Impacts.” Climatic Change 59, no. 1: 5–31.

[ece372412-bib-0004] Bhattarai, B. , R. Chikanbanjar , R. M. Kunwar , R. W. Bussmann , and N. Y. Paniagua‐Zambrana . 2021. “ *Diploknema butyracea* (Roxb.) H.J. Lam. Sapotaceae.” In Ethnobotany of the Himalayas. Ethnobotany of Mountain Regions, edited by R. M. Kunwar , H. Sher , and R. W. Bussmann . Springer. 10.1007/978-3-030-57408-6_84.

[ece372412-bib-0005] Booth, T. H. 2018. “Species Distribution Modelling Tools and Databases to Assist Managing Forests Under Climate Change.” Forest Ecology and Management 430: 196–203.

[ece372412-bib-0006] CBS (Central Beuro of Statistics, Nepal) . 2021. “National Report on Caste/Ethnicity, Language & Religion.” https://censusnepal.cbs.gov.np/results/downloads/casteethnicity.

[ece372412-bib-0007] Chikanbanjar, R. , U. Pun , B. Bhattarai , and R. M. Kunwar . 2021. “Chiuri (*Diploknema butyracea* (Roxb.) HJ Lam): An Economic Tree for Improving Livelihood of Chepang Communities in Makwanpur, Nepal.” Ethnobotany Research and Applications 21: 1–11.

[ece372412-bib-0008] Colwell, R. K. , G. Brehm , C. L. Cardelús , A. C. Gilman , and J. T. Longino . 2008. “Global Warming, Elevational Range Shifts, and Lowland Biotic Attrition in the Wet Tropics.” Science 322, no. 5899: 258–261. 18845754 10.1126/science.1162547

[ece372412-bib-0009] Convertino, M. , G. A. Kiker , R. Muñoz‐Carpena , M. L. Chu‐Agor , R. A. Fischer , and I. Linkov . 2011. “Scale‐and Resolution‐Invariance of Suitable Geographic Range for Shorebird Metapopulations.” Ecological Complexity 8, no. 4: 364–376.

[ece372412-bib-0010] Dhakal, B. 2014. “Development of Chyuri ( *Diploknema butyracea* Roxb) Fruit Biomass Models (A Case Study From Piple Basaha Community Forest of Baglung, Nepal).” 10.13140/RG.2.2.34092.46729.

[ece372412-bib-0011] Duflot, R. , C. Avon , P. Roche , and L. Berges . 2018. “Combining Habitat Suitability Models and Spatial Graphs for More Effective Landscape Conservation Planning: An Applied Methodological Framework and a Species Case Study.” Journal for Nature Conservation 46: 38–47. 10.1016/j.jnc.2018.08.005.

[ece372412-bib-0012] Fox, J. , and S. Weisberg . 2011. “Multivariate Linear Models in R.” An R Companion to Applied Regression Los Angeles Thousand Oaks.

[ece372412-bib-0013] Gurung, G. M. 1989. The Chepangs: A Study in Continuity and Change. Centre for Nepal and Asian Studies, Tribhuvan University, Kathmandu, Nepal.

[ece372412-bib-0014] Hajek, O. L. , and A. K. Knapp . 2022. “Shifting Seasonal Patterns of Water Availability: Ecosystem Responses to an Unappreciated Dimension of Climate Change.” New Phytologist 233, no. 1: 119–125. 34506636 10.1111/nph.17728

[ece372412-bib-0015] Hallegatte, S. , M. Fay , and E. B. Barbier . 2018. “Poverty and Climate Change: Introduction.” Environment and Development Economics 23, no. 3: 217–233.

[ece372412-bib-0016] Joshi, N. C. , A. Chaudhary , and G. S. Rawat . 2018. “Cheura (*Diploknema butyracea*) as a Livelihood Option for Forest‐Dweller Tribe (Van‐Raji) of Pithoragarh, Uttarakhand, India.” International Journal for Environmental Rehabilitation and Conservation 9, no. 1: 134–141. 10.31786/09756272.18.9.1.116.

[ece372412-bib-0017] Joshi, S. R. 2010. “Resource Analysis of Chyuri (*Aesandra butyracea*) in Nepal.” www.medep.org.np.

[ece372412-bib-0018] Ke, S. L. , L. Shu‐Kang , and T. E. Pennington . 1996. “Sapotaceae.” In Flora of China, 205–214. http://www.efloras.org/florataxon.aspx?flora_id=2&taxon_id=10793.

[ece372412-bib-0019] Kerr, J. T. , and S. Z. Dobrowski . 2013. “Predicting the Impacts of Global Change on Species, Communities and Ecosystems: It Takes Time.” Global Ecology and Biogeography 22, no. 3: 261–263.

[ece372412-bib-0020] Koch, R. , J. S. Almeida‐Cortez , and B. Kleinschmit . 2017. “Revealing Areas of High Nature Conservation Importance in a Seasonally Dry Tropical Forest in Brazil: Combination of Modelled Plant Diversity Hot Spots and Threat Patterns.” Journal for Nature Conservation 35: 24–39.

[ece372412-bib-0021] Korner, C. 2007. “The Use of ‘Altitude’ in Ecological Research.” Trends in Ecology & Evolution 22, no. 11: 569–574. 17988759 10.1016/j.tree.2007.09.006

[ece372412-bib-0022] Kunwar, R. M. , K. B. Thapa‐Magar , S. C. Subedi , et al. 2023. “Distribution of Important Medicinal Plant Species in Nepal Under Past, Present, and Future Climatic Conditions.” Ecological Indicators 146: 109879.

[ece372412-bib-0023] Lee, S. K. , and T. D. Pennington . 1996. SAPOTACEAE Shan Lan Ke. In Flora of China (Vol. 150).

[ece372412-bib-0024] Lu, N. , C. X. Jia , H. Lloyd , and Y. H. Sun . 2012. “Species‐Specific Habitat Fragmentation Assessment, Considering the Ecological Niche Requirements and Dispersal Capability.” Biological Conservation 152: 102–109.

[ece372412-bib-0025] Majumdar, K. , B. K. Datta , and U. Shankar . 2012. Establ. Contin. Distrib. 2 (7), 660–666.

[ece372412-bib-0026] Manandhar, N. P. 2002. Plants and People of Nepal. Timber Press.

[ece372412-bib-0027] MoFSC . 2014. Nepal National Biodiversity Strategy and Action Plan 2014–2020, Kathmandu, Nepal.

[ece372412-bib-0028] MoSTE . 2014. Nepal Second National Communication to United Nations Framework Convention on Climate Change, Kathmandu, Nepal.

[ece372412-bib-0029] Muluneh, M. G. 2021. “Impact of Climate Change on Biodiversity and Food Security: A Global Perspective—A Review Article.” Agriculture & Food Security 10, no. 1: 1–25.

[ece372412-bib-0030] Nagendra, H. , R. Lucas , J. P. Honrado , et al. 2013. “Remote Sensing for Conservation Monitoring: Assessing Protected Areas, Habitat Extent, Habitat Condition, Species Diversity, and Threats.” Ecological Indicators 33: 45–59.

[ece372412-bib-0031] NCVST . 2009. Vulnerability Through the Eyes of Vulnerable: Climate Change Induced Uncertainties and Nepal's Development Predicaments. Institute for Social and Environmental Transition (ISET).

[ece372412-bib-0032] Paudel, S. , and K. F. Wiersum . 2002. “Tenure Arrangements and Management Intensity of Butter Tree (*Diploknema butyracea*) in Makawanpur District, Nepal.” International Forestry Review 4, no. 3: 223–230. 10.1505/IFOR.4.3.223.17394.

[ece372412-bib-0033] Pearson, R. G. , and T. P. Dawson . 2003. “Predicting the Impacts of Climate Change on the Distribution of Species: Are Bioclimate Envelope Models Useful?” Global Ecology and Biogeography 12, no. 5: 361–371.

[ece372412-bib-0034] Phillips, H. S. , S. Kharbanda , R. Chen , et al. 2006. “Molecular Subclasses of High‐Grade Glioma Predict Prognosis, Delineate a Pattern of Disease Progression, and Resemble Stages in Neurogenesis.” Cancer Cell 9, no. 3: 157–173. 16530701 10.1016/j.ccr.2006.02.019

[ece372412-bib-0035] Press, J. R. , K. K. Shrestha , and D. A. Sutton . 2000. Annotated Checklist of the Flowering Plants of Nepal. Natural History Museum Publications. http://efloras.org/florataxon.aspx?flora_id=110&taxon_id=242420203.

[ece372412-bib-0036] Profirio, L. L. , R. M. B. Harris , E. C. Lefroy , et al. 2014. “Improving the Use of Species Distribution Models in Conservation Planning and Management Under Climate Change.” PLoS One 9, no. 11: e113749. 10.1371/journal.pone.0113749. 25420020 PMC4242662

[ece372412-bib-0037] Rana, S. K. , H. K. Rana , S. K. Ghimire , K. K. Shrestha , and S. Ranjitkar . 2017. “Predicting the Impact of Climate Change on the Distribution of Two Threatened Himalayan Medicinal Plants of Liliaceae in Nepal.” Journal of Mountain Science 14: 558–570.

[ece372412-bib-0038] Rana, S. K. , H. K. Rana , S. Ranjitkar , et al. 2020. “Climate‐Change Threats to Distribution, Habitats, Sustainability and Conservation of Highly Traded Medicinal and Aromatic Plants in Nepal.” Ecological Indicators 115: 106435.

[ece372412-bib-0039] Ranjitkar, S. , J. Xu , K. K. Shrestha , and R. Kindt . 2014. “Ensemble Forecast of Climate Suit Ability for the Trans‐Himalayan Nyctaginaceae Species.” Ecological Modelling 282: 18–24. 10.1016/j.ecolmodel.2014.03.003.

[ece372412-bib-0040] Rengstorf, A. M. , C. Yesson , C. Brown , and A. J. Grehan . 2013. “High‐Resolution Habitat Suitability Modelling Can Improve Conservation of Vulnerable Marine Ecosystems in the Deep Sea.” Journal of Biogeography 40, no. 9: 1702–1714.

[ece372412-bib-0041] Rijal, A. 2011. “Surviving on Knowledge: Ethnobotany of Chepang Community From Midhills of Nepal.” In Ethnobotany Research and Applications, vol. 9, 1992. Department of Ethnobotany, Institute of Botany, Ilia State University. 10.17348/era.9.0.181-215.

[ece372412-bib-0042] Salick, J. , Z. Fang , and A. Byg . 2009. “Eastern Himalayan Alpine Plant Ecology, Tibetan Ethnobotany, and Climate Change.” Global Environmental Change 19, no. 2: 147–155. 10.1016/j.gloenvcha.2009.01.008.

[ece372412-bib-0043] Shrestha, B. , S. Tsiftsis , D. J. Chapagain , et al. 2021. “Suitability of Habitats in Nepal for *Dactylorhiza hatagirea* Now and Under Predicted Future Changes in Climate.” Plants 10, no. 3: 467. 33801220 10.3390/plants10030467 PMC8000360

[ece372412-bib-0044] Tse‐ring, K. , E. Sharma , N. Chettri , and A. Shrestha . 2010. Climate Change Impact and Vulnerability in the Eastern Himalayas: Synthesis Report. Climate Change Vulnerability of Mountain Ecosystems in the Eastern Himalayas.

[ece372412-bib-0045] Uprety, Y. , and H. Asselin . 2023. “Biocultural Importance of the Chiuri Tree [*Diploknema butyracea* (Roxb.) H. J. Lam]. Chepang Communities of Central Nepal.” Forests 14, no. 3: 479.

[ece372412-bib-0046] Vincze, M. , I. D. Borcia , and U. Harlander . 2017. “Temperature Fluctuations in a Changing Climate: An Ensemble‐Based Experimental Approach.” Scientific Reports 7, no. 1: 254. 28325927 10.1038/s41598-017-00319-0 PMC5428220

[ece372412-bib-0047] Xu, D. , Z. Zhuo , R. Wang , M. Ye , and B. Pu . 2019. “Modeling the Distribution of Zanthoxylum Armatum in China With MaxEnt Modeling.” Global Ecology and Conservation 19: e00691.

[ece372412-bib-0048] Yi, Y. , X. Cheng , Z. Yang , S. Wieprecht , S. Zhang , and Y. Wu . 2017. “Evaluating the Ecological Influence of Hydraulic Projects: A Review of Aquatic Habitat Suitability Models.” Renewable and Sustainable Energy Reviews 68: 748–762.

[ece372412-bib-0049] Yi, Y. J. , Y. Zhou , Y. P. Cai , W. Yang , Z. W. Li , and X. Zhao . 2018. “The Influence of Climate Change on an Endangered Riparian Plant Species: The Root of Riparian Homonoia.” Ecological Indicators 92: 40–50.

[ece372412-bib-0050] Zheng, H. , G. Shen , L. Shang , et al. 2016. “Efficacy of Conservation Strategies for Endangered Oriental White Storks ( *Ciconia boyciana* ) Under Climate Change in Northeast China.” Biological Conservation 204: 367–377.

[ece372412-bib-0051] Zu, K. , Z. Wang , X. Zhu , et al. 2021. “Upward Shift and Elevational Range Contractions of Subtropical Mountain Plants in Response to Climate Change.” Science of the Total Environment 783: 146896. 33866165 10.1016/j.scitotenv.2021.146896

